# A Rare Case of Vancomycin-Induced Drug Reaction With Eosinophilia and Systemic Symptoms (DRESS) Syndrome

**DOI:** 10.7759/cureus.73088

**Published:** 2024-11-05

**Authors:** Deobrat Mallick, Nayanjyoti Kaushik, Lokesh Goyal, Deepak Chandramohan, Prathap Simhadri, Prabhat Singh

**Affiliations:** 1 Internal Medicine, CHRISTUS Spohn Hospital, Corpus Christi, Corpus Christi, USA; 2 Electrophysiology, Cardiology, University of Iowa Hospitals and Clinics, Iowa City, USA; 3 Hospital Medicine, CHRISTUS Spohn Hospital, Corpus Christi, Corpus Christi, USA; 4 Nephrology, University of Alabama, Birmingham, USA; 5 Internal Medicine/Nephrology, AdventHealth Graduate Medical Education/Florida State University College of Medicine, Daytona Beach, USA; 6 Nephrology, CHRISTUS Spohn Hospital, Corpus Christi, Corpus Christi, USA

**Keywords:** drug reaction with eosinophilia and systemic symptoms (dress), drug reaction with eosinophilia and systemic symptoms (dress) syndrome, generalized skin rash, vancomycin, vancomycin infusion

## Abstract

Drug reaction with eosinophilia and systemic symptoms (DRESS) is a rare but potentially life-threatening adverse drug reaction characterized by extensive skin rash in association with hematological abnormalities, including eosinophilia and atypical lymphocytosis, lymphadenopathy, fever, and extensive visceral organ involvement. Here, we presented a rare case of vancomycin-induced DRESS syndrome in a male who was treated with IV vancomycin for a brain abscess.

## Introduction

Drug reaction with eosinophilia and systemic symptoms (DRESS) syndrome is a rare drug reaction, estimated to have an incidence between one in 1,000 and one in 10,000 exposures. It is considered a type IV allergic reaction (T-cell-mediated hypersensitivity reaction) [1-5]. The exact pathogenesis is not fully understood.

Evidence suggests two main pathogenic mechanisms for DRESS syndrome: drug-specific immune response and human herpes viral reactivation with antiviral immune response.

Antiepileptic drugs are the most associated with DRESS syndrome, but the link with vancomycin is less well-established.

Disease courses are typically prolonged with heterogeneous clinical presentations. Despite the cessation of the enflaming drug, the disease may continue to progress. The latency between the introduction of the drug and the onset of the disease is prolonged and typically takes between two and eight weeks [6]. A commonly observed phenomenon with DRESS syndrome is the reactivation of latent viruses [7-9]. Reactivation of human herpesviridae viruses (e.g., HHV-6, HHV-7), Epstein-Barr virus (EBV), and cytomegalovirus (CMV) occur in up to 75% of patients [10-14].

## Case presentation

**Figure 1:**
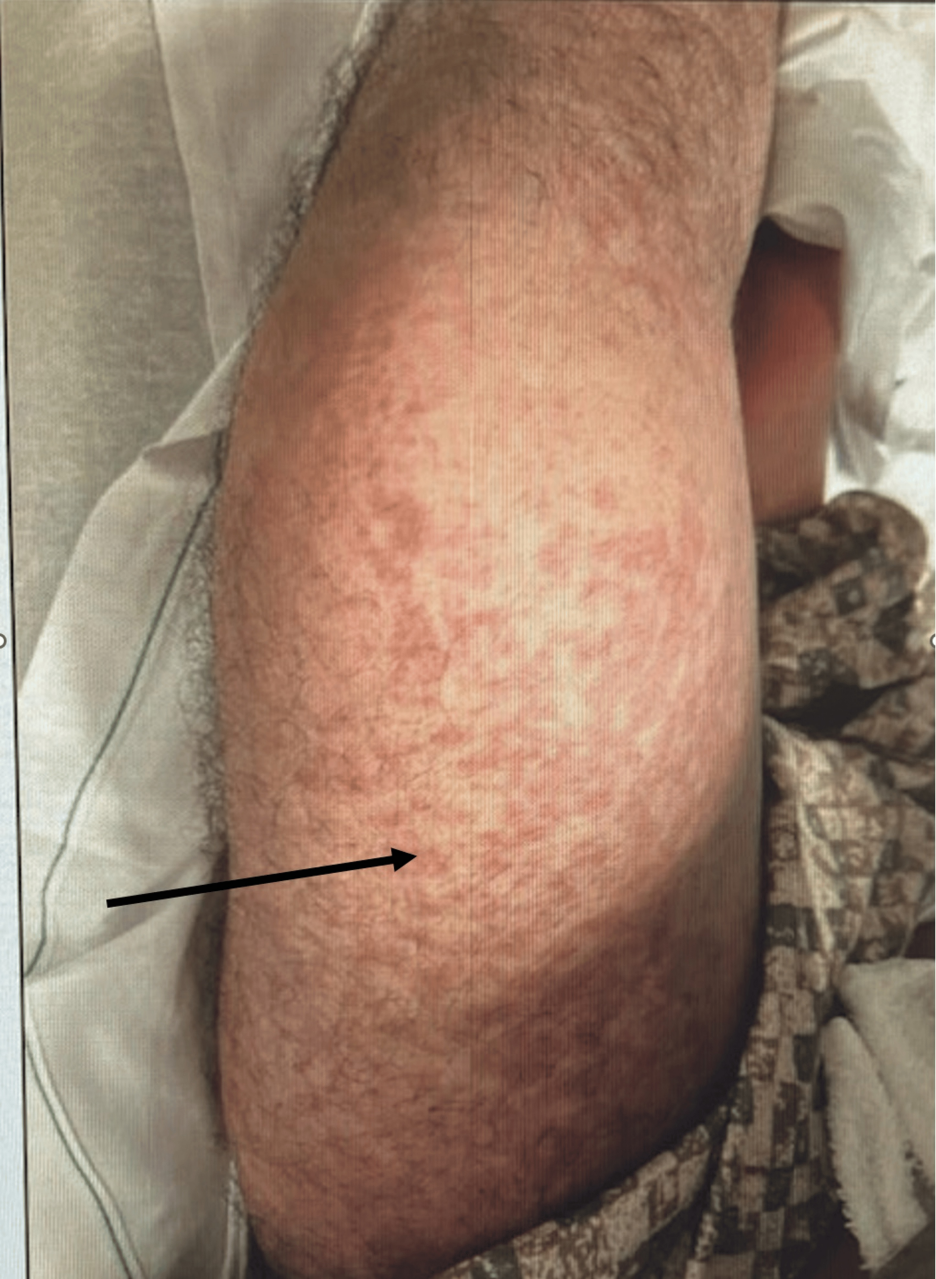
Skin rash of DRESS syndrome DRESS: Drug reaction with eosinophilia and systemic symptom

**Figure 2:**
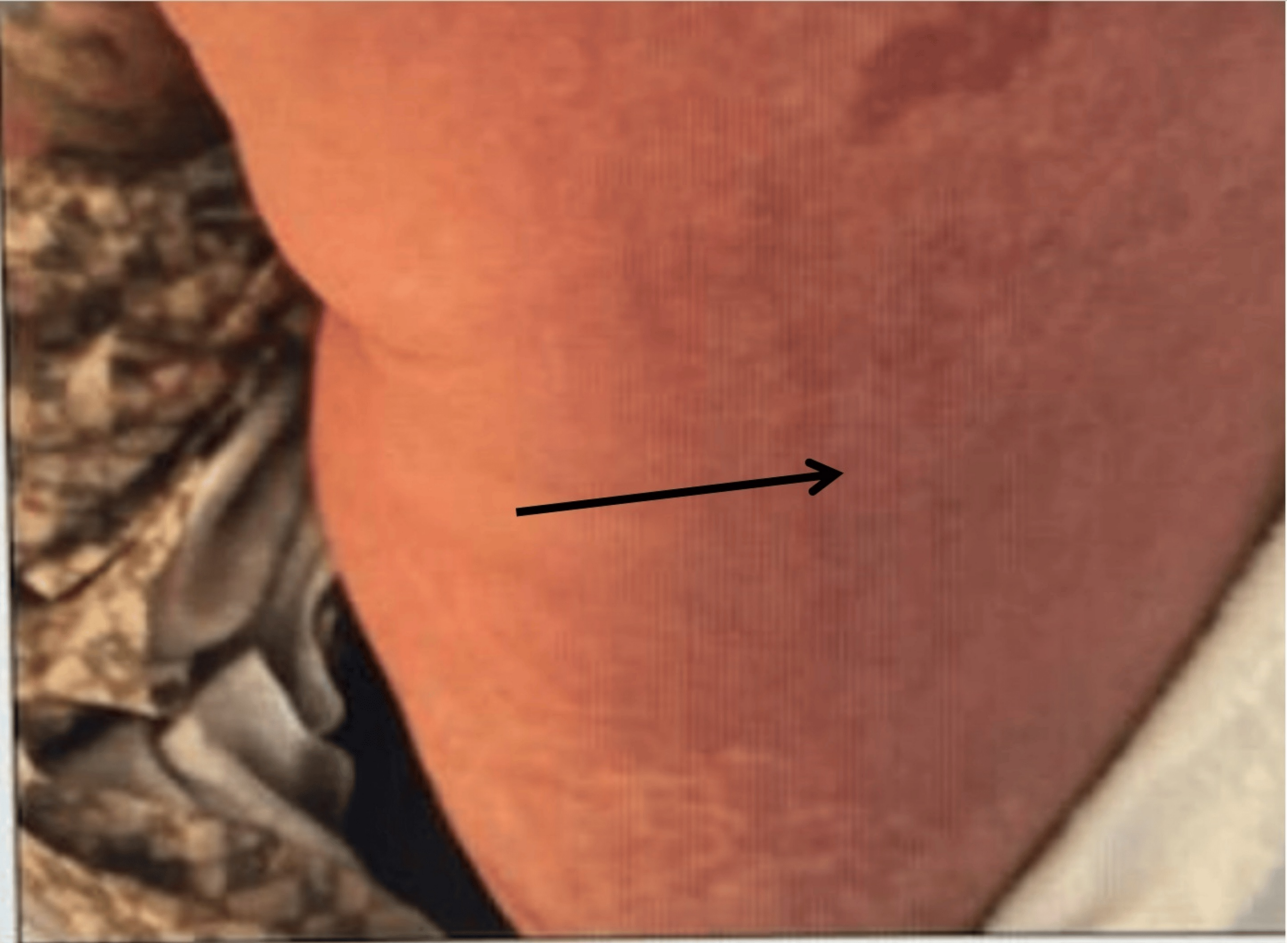
Skin rash of DRESS syndrome DRESS: Drug reaction with eosinophilia and systemic symptom

A 44-year-old gentleman, with no significant past medical history, was recently diagnosed with a brain abscess and was discharged home on a six-week course of IV vancomycin and ceftriaxone. The patient was self-administering the antibiotic at home. The patient was doing well and tolerated the antibiotic well until about four weeks after being discharged from the hospital when he suddenly developed a widespread maculopapular rash and fever. This prompted the patient to seek urgent medical attention, and he presented to the emergency room for evaluation. Skin examination was notable for diffuse erythematous papules that initially appeared on his neck and spread down his torso to bilateral upper and lower extremities (Figures 1-2).

ER physician suspected "red man" syndrome associated with vancomycin. The vancomycin trough was also high. The patient received a dose of antihistamine (diphenhydramine) and H2 blocker (famotidine), with some improvement in the patient's rash. The dose of vancomycin was reduced, and the patient was discharged from the ER on oral H1 and H2 blockers. The patient was advised to administer vancomycin slowly to avoid "red man" syndrome. However, in the next couple of days, the patient's symptoms continued to get worse with widespread extensive maculopapular rash throughout the torso, face, head, neck, and thighs, with swelling and severe itching, as well as high-grade fever. When the patient returned to the ER for re-evaluation, he showed no signs of tongue swelling or airway compromise. Blood work was suggestive of severe eosinophilia and moderate leukocytosis with elevated liver enzymes (Table 1).

**Table 1:** Lab test results of the patient

Lab Test	Before initiation of antibiotic (vancomycin)	At the time of onset of symptom (4 weeks post antibiotic)	Seven days post methylprednisolone treatment
Hemoglobin (g/dL)	12.4	10.5	10.6
WBC (X1000/uL)	11.8	28.5	19.7
Neutrophils (%	77.8	64.9	56.7
Lymphocytes (%)	14.6	7.8	25.6
Eosinophils (%)	0.2	20.0	5.0
Absolute eosinophils count (/mm^3^)	23	5700	985
Atypical lymphocytes	Absent	Present	Present
Platelets (/mm^3^)	387	499	334
Serum bilirubin (mg/dL)	0.5	0.2	0.3
ALT (iu/L)	39	186	72
AST (iu/L)	19	13	16
Alkaline phosphatase (iu/L)	53	50	47
Serum creatinine (mg/dL)	1.0	1.1	1.1
BUN (mg/dL)	11	18	26

Serology for hepatitis A virus (HAV), hepatitis B virus (HBV), hepatitis C virus (HCV), human herpesvirus (HHV), and cytomegalovirus (CMV), mycoplasma, chlamydia, and anti-nuclear antibody (ANA) were negative. All antibiotics were discontinued.

Based on the patient's clinical symptoms and the RegiSCAR scoring system (Table 2). The patient was diagnosed with DRESS syndrome and was promptly started on IV methylprednisolone. The patient's rash and swelling continued to get worse for the next couple of days, but then symptoms gradually began to improve. The ID physician gradually reintroduced antibiotics, one by one, except vancomycin (which was replaced with linezolid), to complete the antibiotic course for his brain abscess. The patient tolerated the antibiotics quite well without any hypersensitivity reaction. MRI of the brain showed significant improvement in the size of the abscess. The patient was discharged after six weeks of tapering his steroid dose. At the time of discharge, the patient had a 90% improvement in rash and complete normalization of eosinophil count.

**Table 2:** RegiSCAR scoring system for the diagnosis of DRESS syndrome The Registry of Severe Cutaneous Adverse Reactions (RegiSCAR) scoring system for the diagnosis of DRESS syndrome [9,15].

SCORE	-1	0	1	2
Fever >38.5	No/unknown	Yes		
Lymphadenopathy:>1 cm, at least 2 sites		No/unknown	Yes	
Eosinophilia		No/unknown	≥0.7 × 109 or ≥10% if leukopenia	≥1.5 × 109
Atypical lymphocytes		No/unknown	Yes	
Skin Rash suggest DRESS: ≥2 facial edemas, purpura, infiltration, desquamation	No	Unknown	Yes	
Rash Extent >50%		No/unknown	Yes	
Biopsy suggestive of DRESS	No	Yes/unknown		
Disease duration ≥15 days	No/unknown	Yes		
Organ involvement		No	1 organ involved	≥2 organ involved
Exclusion of other causes: 3 of the following tests are performed and are negative: HAV, HBV, HCV, mycoplasma, chlamydia, ANA		No/unknown	Yes	

## Discussion

DRESS syndrome is a rare but potentially life-threatening drug-induced hypersensitive reaction associated with multisystem involvement. Among all the cutaneous adverse drug reactions in hospitalized patients, DRESS accounts for 10-20% of cases [15-17]. Risk varies from drug to drug (Table 3). In approximately 80% of cases, a clear drug trigger can be identified, but the strength of drug causality is less clear in the remaining 10-20% [18-21]. Most of the cases of DRESS are caused by high-risk drug groups (Table 3).

**Table 3:** High- and low-risk drugs associated with DRESS This is the list of high- and low-risk drugs associated with DRESS [7,8,16-19].

High-risk drugs
Antiepileptic agents: Lamotrigine, Carbamazepine, Piperacillin, Phenytoin, Phenobarbital, Oxcarbazepine
Sulfonamides: Trimethoprim-sulfamethoxazole, Sulfasalazine, Dapsone, Sulfadiazine
Antituberculosis agents: Isoniazid, Rifampicin, Ethambutol, Pyrazinamide
Vancomycin, Allopurinol, Minocycline, Nevirapine, Mexiletine

Low-risk drugs
Beta-lactams: Ampicillin, Amoxicillin, Piperacillin
Other Drugs: Olanzapine, Fluoxetine, NSAIDs, Imatinib, Sorafenib, Vemurafenib

Vancomycin is an uncommon cause of DRESS syndrome, but there is a significant rise in incidence due to the increased use of vancomycin in hospitalized patients in the United States. However, some patients may be genetically predisposed due to HLA variation [16,22-24].

Vancomycin is accountable for about 2/3 of the antibiotic-associated DRESS syndrome [25].

From drug initiation to onset of the reaction typically ranges from two to eight weeks but may be shorter in some cases of beta-lactam antibiotic and iodine contrast media. Cutaneous manifestation is the most common symptom. It begins as an erythematous maculopapular eruption, which may progress to coalescing erythema. Symmetrically distributed on the trunk and extremities, it usually involves more than 50% of the body's surface area. Facial edema is present in most of the cases (>70%) and may be a useful clue to the diagnosis [9,26]. Pruritus may also be present in many cases. Up to 50% of cases of mucosal involvement may be seen, but they are typically mild compared to Steven-Johnson syndrome/toxic epidermal necrolysis. Systemic symptoms, such as a fever of more than 38.5 degrees Fahrenheit, occur in up to 90% of cases. Up to 65% of patients have lymphadenopathy. Hematological abnormality includes eosinophilia (82-95%), leukocytosis (95%), neutrophilia (78%), lymphocytosis (25 to 52%), monocytosis (69%), atypical lymphocytes (35-67%), lymphocytopenia (45%), and thrombocytopenia (25%). 

Eosinophilia can be absent in a significant subset of patients and is not necessary to diagnose DRESS. At least one internal organ is involved in approximately 90% of patients. The liver is the most common organ involved in DRESS syndrome, affecting 50-90% of patients [27-29]. Renal involvement ranges from proteinuria to kidney failure, needing dialysis [27,28]. Pulmonary involvement can occur in up to 30% of patients and may include acute interstitial pneumonitis, lymphocytic interstitial pneumonia, pleuritis, pleural effusion, and acute respiratory distress syndrome (ARDS) [30-32]. Around 2-20% of cases can have cardiac involvement with myocarditis, which is a poor prognostic factor [33,34]. Cardiac involvement presents with hypotension, tachycardia, chest pain, left ventricular dysfunction, and abnormal EKG. Nervous system involvement has been seen in 2-8% of cases, including Bell's palsy, peripheral neuropathy, aseptic meningitis, vasculitis, and encephalitis [7,35-38].

Gastrointestinal (GI) involvement, although uncommon, may cause GI bleed, esophagitis, colitis, cholecystitis, or intestinal perforation [39-42].

The severity of DRESS ranges from mild or without organ involvement to severe with multiorgan involvement and life-threatening conditions (Table 4).

**Table 4:** Severity of DRESS syndrome based on the extent of organ involvement Source: [20] DRESS: Drug reaction with eosinophilia and systemic symptoms

Mild DRESS	Moderate DRESS	Severe DRESS
With or without modest liver involvement, such as elevation of liver transaminases <4 times the upper limit of normal, in the absence of clinical, laboratory, or imaging evidence of renal, pulmonary, or cardiac involvement.	Hemoglobin 7 to 10 g/dL and/or neutrophils 500 to 1500/dL	Major cytopenia (hemoglobin <7g/dL, neutrophils <500/dL, platelets <50,000/dL)
	Platelets 50,000 to 100,000/dL	Pancytopenia
	Creatinine >26.4 mmol/L or 1.5 times the upper limit of normal	Hemophagocytosis
	Liver enzymes 4 to 15 times the upper limit of normal and/or alkaline phosphate three to five times the upper limit of normal	Rapidly progressive and/or major eosinophilia
		Rapidly progressive and/or major atypical lymphocytosis
		Major kidney failure or rapidly progressive oligo/anuria
		Liver enzymes >15 times upper limit normal and alkaline phosphatase >5 times upper limit normal and/or factor V <50 percent
		Involvement of any other organ (e.g., heart, lungs, nervous system)/multiorgan involvement

DRESS is a dynamic process, and it is essential to understand that characteristic features are not all present concurrently. Although uncommon, it is possible that a patient may have no or minimal skin rash (<5%), absent eosinophilia, and mild or no systemic symptoms or organ involvement [9,27]. In such cases, a high degree of suspicion and clinical judgment is required to diagnose DRESS syndrome. The Registry of Severe Cutaneous Adverse Reactions (RegiSCAR) scoring system (Table 2) is the most widely used criteria to confirm or exclude the diagnosis of DRESS. Thymus and activation-regulated chemokine (TARC) levels may be used as a diagnostic and activity biomarker because they are significantly higher in DRESS than in other drug hypersensitivity skin involvement. Skin biopsy is only done to rule out other conditions that may mimic DRESS. Histopathological features of DRESS are heterogeneous and nonspecific [3]. Two important factors that should be considered in the assessment of drug causality for DRESS are prolonged latency (two to eight weeks after drug exposure) and exposure to high-risk drugs.

Mainstay of the treatment is the identification and withdrawal of the causative drugs and supportive treatment. 

Mild DRESS treatment includes high- or super-high-potency topical corticosteroids two to three times per day until resolution of skin eruption.

For moderate DRESS treatment, options include super-high-potency topical corticosteroids or a course of systemic steroids.

Severe DRESS treatment options include oral corticosteroid with supportive treatment. Prednisone or prednisone equivalents are administered at a dose of 0.5 to 1 mg/kg/day until clinical improvement and laboratory parameter normalization. Steroids should be tapered slowly over eight to 12 weeks. Patients’ refractory to corticosteroids should be treated with oral cyclosporine. Cyclosporine dose is 3-5 mg/kg/day in two divided doses for seven days, followed by a taper over the next seven to 14 days.

## Conclusions

There is a significant rise in the incidence of vancomycin-induced DRESS syndrome due to the increased use of vancomycin in hospitalized patients. The severity of DRESS could vary from mild to severe with life-threatening multiorgan involvement. Diagnosis of DRESS is often delayed, leading to complications and even death. It is important to identify this condition early and treat it promptly to reduce morbidity and mortality significantly. A case report such as this one brings the attention of its readers to the rare side effects of this commonly prescribed antibiotic.
